# The effect of reducing the incidence of gastrointestinal complications in patients treated with aspirin, referred to Imam Hospital, of Ahvaz, Iran

**DOI:** 10.1016/j.dib.2017.09.065

**Published:** 2017-10-02

**Authors:** Abdalrahim Masjedizade, Mohammad Javad Mohammadi, Saeed Yazdankhah, Atefeh Roumi, Arman Shahriari, Sahar Geravandi

**Affiliations:** a Research Center for Infectious Diseases of Digestive System, Alimentary Tract Research Center, Ahvaz Jundishapur University of Medical Sciences, Ahvaz, Iran; b Abadan school of Medical Sciences, Abadan, Iran; c Asadabad school of Medical Sciences, Asadabad, Iran

**Keywords:** HPE, Helicobacter pylori eradicate, Imam H, Imam Hospital, GC, gastrointestinal complications, Helicobacter pylori, Aspirin, Patients, Imam Hospital, Iran

## Abstract

This research contains data which were obtained during the analysis of treated patients with aspirin who were referred to Imam Hospital (Imam H) of Ahvaz, Iran; and the effect of this type of treatment (Helicobacter pylori eradicate (HPE)) on reducing the incidence of gastrointestinal complications. Studies have shown that taking aspirin in patients with Helicobacter pylori infection is associated with a decreasing risk of gastrointestinal bleeding (O'connor et al., 2013) [1]. In this study, 165 patients with positive helicobacter pylori infection test were chosen among those who were referred to Imam H. In this paper, the effects of sex, smoking, renal failer, diabetes, age, blood pressure and aspirin consumption have been studied (Fletcher et al., 2010) [2]. After completion of the observations and records of patient's medical records, the obtained coded data were fed into EXCELL. Data analysis was performed, using SPSS 16.

**Specifications Table**

**Table:** 

Subject area	*Medicine, clinical research*
More specific subject area	*Effect of helicobacter pylori eradicate on reducing the incidence of gastrointestinal complications*
Type of data	*Table, figure*
How data was acquired	*Functional clinical assessment of the patients with positive helicobacter pylori infection test*
Data format	*Raw, analyzed, Descriptive and statistical data*
Experimental factors	– *Sample consisted of patients with positive helicobacter test who were referred to Imam Hospital.* – *After* referral *the* patients *with positive helicobacter test,* demographic *data effect of aspirin on reducing the incidence of gastrointestinal complications by observations and patient's medical records were completed.* – *In this paper, the effects of* sex*, smoking, renal failer, diabetes, age, blood pressure and aspirin consumption have been studied.*
Experimental features	*Helicobacter pylori is one of the* most *common gastrointestinal complications.*
Data source location	*Ahvaz, Iran*
Data accessibility	*Data is included in this article.*

**Value of the data**

• These data describe effective factors of Helicobacter pylori eradicate in patients with positive helicobacter test and helps with educating the community for the decrease and prevention of gastrointestinal complications.
• Due to the importance of the effect of Helicobacter pylori eradicate in treated patients with aspirin and clopidogrel and reducing the incidence of gastrointestinal complications are discussed in this article.
• The results showed that HPE and treated with aspirin and clopidogrel can be useful for gastrointestinal complications on patients.
• The results of this study can be used to develop a treatment method to decrease gastrointestinal complications in patients.
• Results are also important for patients with gastrointestinal patients especially patients with positive helicobacter pylori infection test who are referred to Hospital.

## 1 Data

Table 1 represents demographic characteristics of patients with positive helicobacter pylori infection who were referred to Imam Khomeini Hospital of Ahvaz, Iran during 2013; used for description of experiments. Table 2 shows data for effective factors of reducing the incidence of gastrointestinal complications in patients treated with aspirin referred to Imam Hospital, Ahvaz, Iran. Based on the result of this study among all factors, highest score was related to aspirin consumption, smoking and blood pressure. The results showed that the most important causes of decreasing gastrointestinal complications (GC) in patients with positive helicobacter pylori infection were related to the aspirin consumption (*P* = 0.0002). Factors related to GC were sex, smoking, renal failer, diabetes, age, blood pressure and aspirin consumption *P* = 0.074, *P* = 0.03, *P* = 0.33, *P* = 0.94, *P* = 0.9, *P* = 0.094 and *P* = 0.0002, respectively. Based on the results, in order to prevent and treat the gastrointestinal complications should be avoided from the provocative activities which mentioned above. Also, according to the results, taking aspirin can be very useful.

**Table 1:** Demographic characteristics of patients with positive helicobacter pylori infection referred to Imam Khomeini Hospital, Ahvaz, Iran during 2013.

**Parameter**	**Characteristics**	**Number (In percent)**
Age group	20–29	9 (5.45%)
	30–39	18 (10.9%)
	40–49	86 (52.12%)
	50–59	38 (23.04%)
	More than 60	14 (8.49%)
Sex	Men	91(55.15%)
	Women	74(44.85%)
Diabetes	Yes	68(41.21%)
	No	97(58.79%)
Blood pressure	Yes	74(44.85%)
	No	91(51.15%)

**Table 2:** Ranking of factors affecting the reducing the incidence of gastrointestinal complications in patients treated with aspirin.

**Factors**	***P* value**
Sex	0.074
Age	0.9
Renal failer	0.33
Diabetes	0.94
Blood pressure	0.094
Smoking	0.03
Aspirin	0.0002

## 2 Experimental design, materials and methods

### 2.1 Study area description

**Fig. 1:**
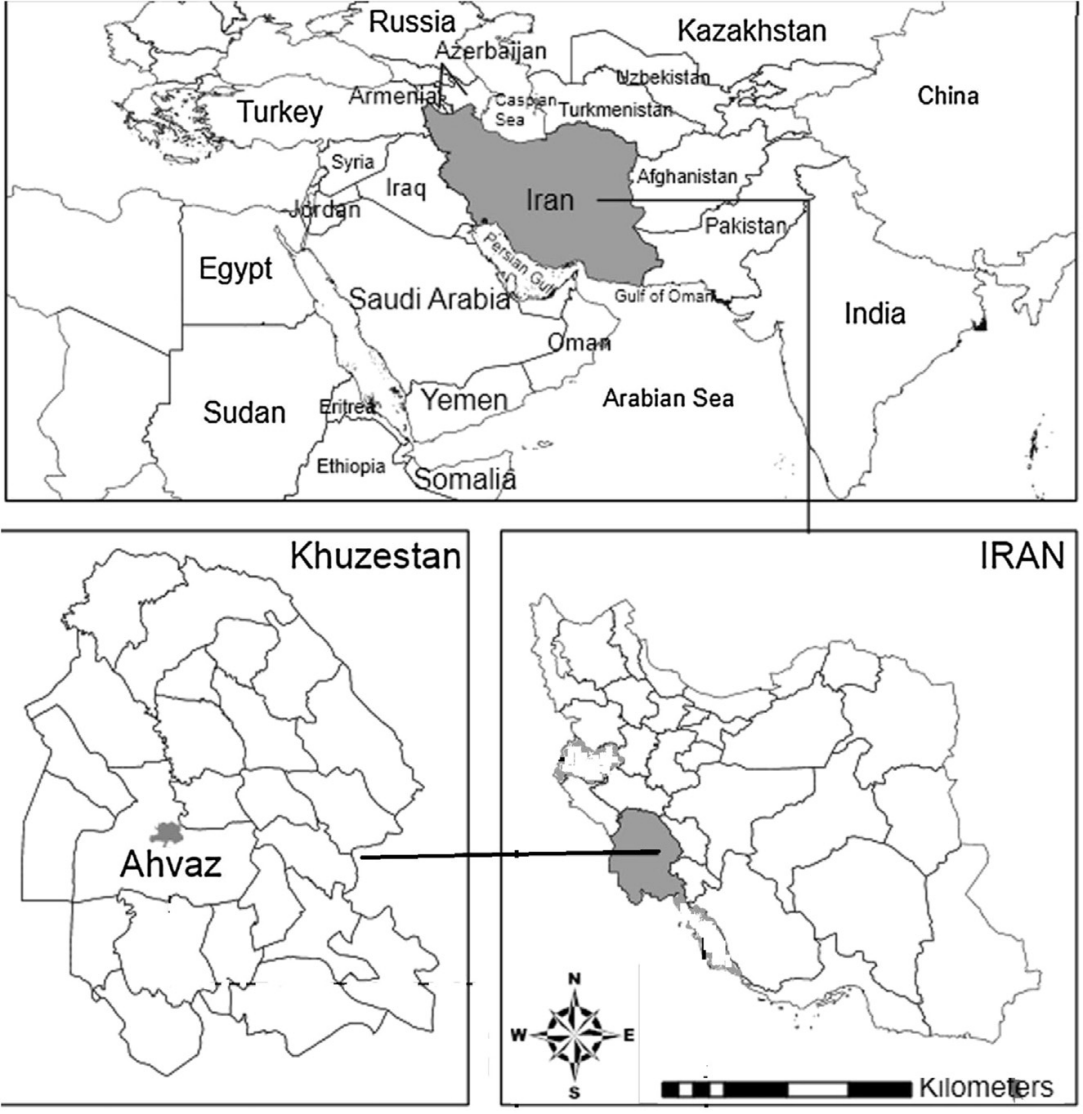
Location of Ahvaz city (Imam teaching hospital).

This clinical trial study was conducted during 2013 at Imam teaching hospital of Ahvaz (a tertiary-care hospital) with 900 beds approximately, in the southwest of Iran. Ahvaz megacity Located in south west of Iran in between 48° and 49°29′ east of the Greenwich meridian and, 31°and 45′ minutes north of the equator. It's the capital city of Khuzestan province, with an area of 140 square kilometers [3], [4], [5], [6], [7]. Ahvaz is Located in the southwest of Iran (see Fig. 1).

### 2.2 Experimental design, materials and methods

165 patients with positive helicobacter pylori infection test referred to Imam teaching hospital with a double-blind randomized into two treatment groups and the control group were studied. 76 and 89 patients were placed in the control and treatment groups, respectively. In this study, data were gathered from the patients with positive helicobacter pylori infection referred to Imam Khomeini H during 2013 as well as a functional clinical assessment including the demographic data (e.g. age, sex) and effective factors of reducing the incidence of gastrointestinal complications in patients who were treated with aspirin including renal failer, diabetes, blood pressure, smoking, smoking and aspirin consumption [2], [8], [9]. Then, the coded data were entered to EXCEL and the analysis was performed, using SPSS 16. All risk factors of the effect of helicobacter pylori eradicate in patients who were treated with aspirin and clopidogrel and reducing the incidence of GC were analyzed. The data were analyzed, applying descriptive and statistical tests including independent *t*-test and chi-square.

### 2.3 Ethics approval and consent to participate

The study was preceded by approval of the Research Ethics Committee of Ahvaz Jundishapur University of Medical Sciences (AJUMS) (protocol number: IR.AJUMS.REC.1393. 305).
