# Supplementary material for: The effect of reducing the incidence of gastrointestinal complications in patients treated with aspirin, referred to Imam Hospital, of Ahvaz, Iran
Source: Data Brief. 2017 Oct 2;15:478–82. doi: 10.1016/j.dib.2017.09.065 (PMC5647466; doi:10.1016/j.dib.2017.09.065)
Supplement: Supplementary file 1 — Transparency document [file mmc1.doc]

**The effect of reducing the incidence of gastrointestinal complications in patients treated with aspirin, referred to Imam Hospital, of Ahvaz, Iran**

**Conflicts of Interest**

Authors have no conflicts of interest.

**Acknowledgment**

The authors would like to thank Ahvaz Jundishapur University of Medical Sciences for providing financial supported by grant: (RDC-9305) of this research.

**Funding/Support**

This study was supported by Ahvaz Jundishapur University of Medical Sciences for providing financial supported by grant: (RDC-9305) of this research.
